# Case report - coronary vasospasm in transplanted heart: a puzzling phenomenon

**DOI:** 10.1186/s12872-019-01280-8

**Published:** 2019-12-19

**Authors:** M. Pagnoni, J. Regamey, J. Adjedj, G. Rogati, O. Muller, P. Tozzi

**Affiliations:** 1 Service de chirurgie cardiaque, Département cœur-vaisseaux, Centre Hospitalier Universitaire Vaudois, Rue du Bugnon 46, 1011 Lausanne, Switzerland; 2 Service de cardiologie, Département cœur-vaisseaux, Centre Hospitalier Universitaire Vaudois, Rue du Bugnon 46, 1011 Lausanne, Switzerland; 3 Transplant coordination unit, Département cœur-vaisseaux, Centre Hospitalier Universitaire Vaudois, Lausanne, Switzerland

**Keywords:** Coronary artery spasm, Heart transplant, Ventricular tachycardia, Myocardial ischemia, Cardiac allograft vasculopathy, Endothelial dysfunction

## Abstract

**Background:**

Coronary artery spasm (CAS) is an underdiagnosed disease especially in heart transplant patients, and in those patients the etiology and pathophysiology remain largely unknown, although it has been associated with cardiac allograft vasculopathy or graft rejection.

**Case presentation:**

We report the case of a heart-transplant patient whose cardiac graft experienced two coronary vasospasms: the first before transplantation, and the other at one-month of a postoperative course complicated by primary graft failure.

**Conclusion:**

Our case illustrates that a transplanted heart predisposed with coronary vasospasm may suffer from early relapse in the recipient despite of complete post-surgical autonomic denervation. Exacerbated endothelial dysfunction of the donor heart after transplant, with the addition of systemic factors in the recipient may be involved in the genesis of this puzzling phenomenon.

## Background

Coronary Artery Spasm (CAS) is a condition caused by the vasoconstriction of the epicardial coronary arteries, leading to myocardial ischemia that can play a role in various conditions, from rest angina to acute coronary syndrome. Several responsible mechanisms have been reported: abnormal autonomic nervous system, endothelial dysfunction, hyperreactivity of the coronary smooth muscle and, as recently demonstrated, specific anatomy of the coronary artery and inflammation of perivascular component, with a fundamental role played by inflammation of coronary adventitia and perivascular adipose tissue through Rho-kinase activation, one of the main molecular mediator in the mechanisms of coronary artery spasm in animals and humans [1–3].

CAS in heart transplant has previously been described to be rare [4]. Nevertheless, various degree of asymptomatic vasospasm may be observed during routine coronary angiography in transplanted hearts [5, 6], which suggests that only the most severe episodes may have overt clinical consequences, such as symptomatic ventricular arrhythmias, high-degree atrio-ventricular block, syncope, cardiac arrest, or even myocardial infarction in case of prolonged ischemia. Denervation of the transplanted heart explains the absence of angina in most cases, although some patients may be symptomatic due to late graft reinnervation [4, 7–10].

Since autonomic innervation is not necessary to develop CAS in heart transplant [4, 10], pathophysiology remains poorly understood and hypothetic. Endothelial damage caused by circulating neurohormonal, immunological or metabolic factors, is suspected to be the main trigger by altering vascular smooth muscle response. Peri-operative ischemia-reperfusion injuries, as well as preexisting preclinical atherosclerosis in the donor heart may also promote endothelial dysfunction and be responsible for abnormal vascular reactivity [8, 11, 12]. Moreover, CAS has been associated with acute rejection episodes and cardiac allograft vasculopathy (CAV), a form of accelerated atherosclerosis characterized by diffuse circumferential neointimal proliferation, which is recognized as the most important cause of long-term death in heart transplant patients. Therefore, CAS has been pointed out as a marker of poor prognosis in heart transplantation and may actually be the first manifestation of CAV [7, 13].

For the diagnosis, ECG changes in ST-T segments during chest symptoms, including good response to nitroderivates, is very important, but the gold standard is represented by Spasm Provocating Tests (SPT), using either Acethylcoline (Ach), either Ergonovine maleate (EM) or a combination of both (sequential SPT): the criteria of positivity is a > 90% transient stenosis of a coronary artery with signs/symptoms of myocardial ischemia [14, 15, 16].

Our case illustrates that a transplanted heart predisposed with coronary vasospasm may suffer from early relapse in the recipient despite of complete post-surgical autonomic denervation.

## Case presentation

**Fig. 1:**
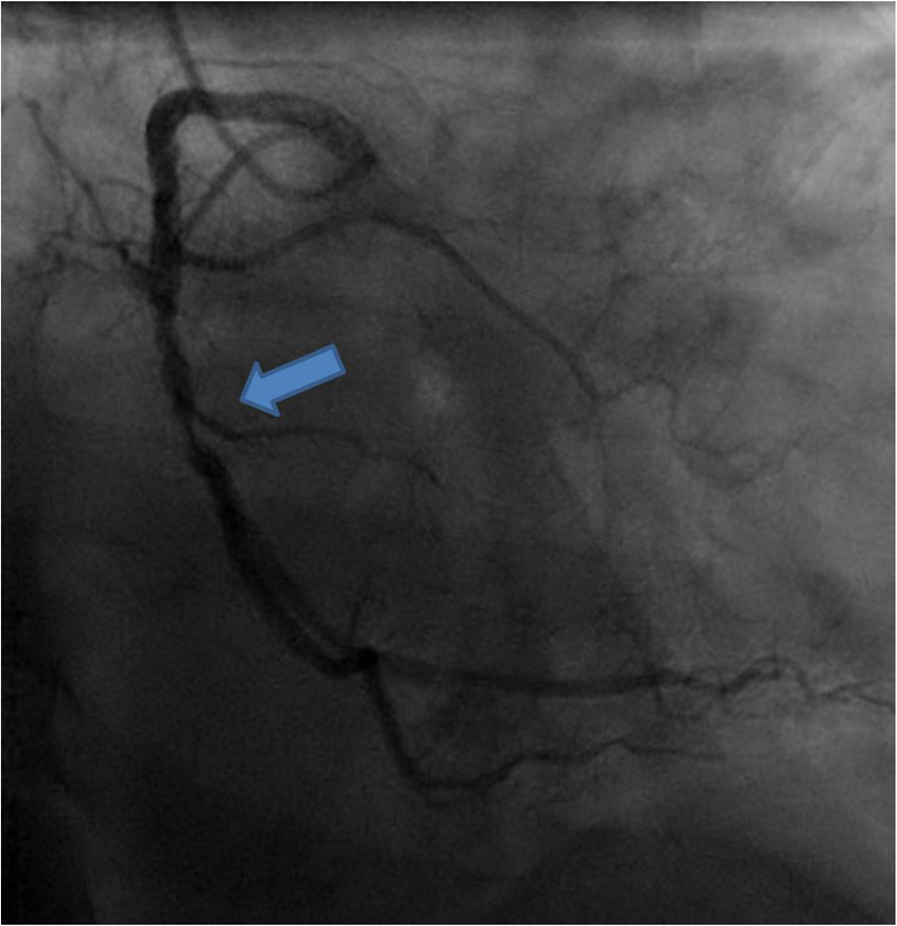
Angiography in the donor heart. Arrow indicates critical stenosis on RCA

A 46 years old man with end-stage hypertrophic cardiomyopathy and electrical storm underwent urgent orthotopic heart transplantation: the donor was a 54 years old woman, known for diabetes, nicotine consumption, morbid obesity, combined ventilation disorder with restriction due to obesity hypoventilation syndrome and suspected chronic obstructive pulmonary disease, and history of thrombophilia (activated protein C resistance) with recurrent deep vein thrombosis and pulmonary embolism in cerebral death due to brain hemorrhage. Pre-transplant cardiac workup showed a 90% stenosis in the middle right coronary artery (Fig. 1), with normal LVEF, and no segmental wall-motion abnormalities or valvulopathy.

The technically uneventful transplant was completed with a venous coronary artery bypass graft (CABG) on the right coronary artery (RCA), with a total ischemic time of 191 min. After cross-clamp removal and appropriate induction therapy with methylprednisolone 500 mg IV, the heart showed severe global biventricular failure with severe functional mitral regurgitation. In the absence of preformed donor specific HLA antibodies in favor of an acute humoral rejection, primary graft failure was suspected, and mechanical hemodynamic support was immediately initiated with a central veno-arterial extracorporeal membrane oxygenation (ECMO) and intra-aortic balloon pump (IABP), and high dose of cathecolamines (Noradrenaline up to 30 mcg/min) A relatively low troponin release was observed during the first 24 h post-operative (peak at 2386 ng/l), favoring the hypothesis of myocardial stunning over necrosis.

**Fig. 2:**
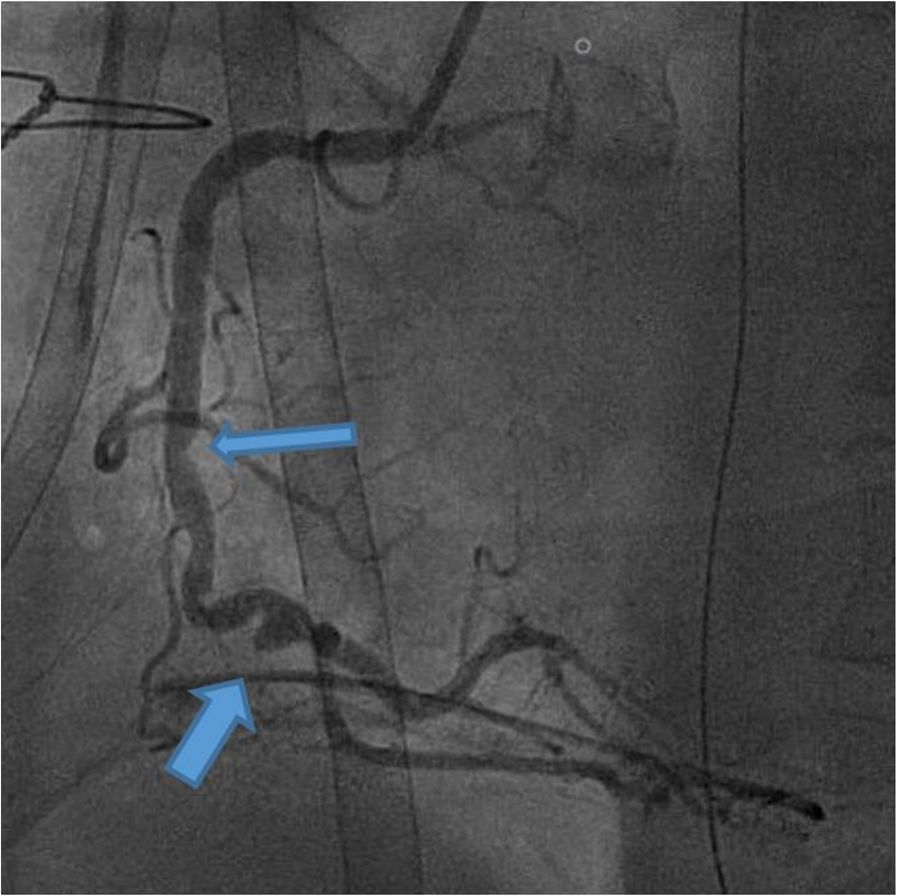
Angiography at day 1: Venous bypass is occluded: broad arrow indicates the anastomotic stump. Thin arrow indicated the < 50% stenosis

A cardiac tamponade on post-operative day 1 led to surgical revision. The intra-operative status was noteworthy for an occlusion of the venous CABG on the RCA. A coronary angiogram was urgently performed in attempt to treat the stenosis in the native vessel. Surprisingly, only a < 50% stenosis could be seen the mid RCA (Fig. 2). It was then noticed that the angiogram performed in the donor had not been preceded by the administration of nitroglycerine, which retrospectively spoke for a severe localized vasospasm of the mid RCA at the site of an atherosclerotic plaque. No further intervention was performed and the ECMO could finally be weaned on day 9, after full recovery of the LV function and moderate persistent RV dysfunction.

**Fig. 3:**
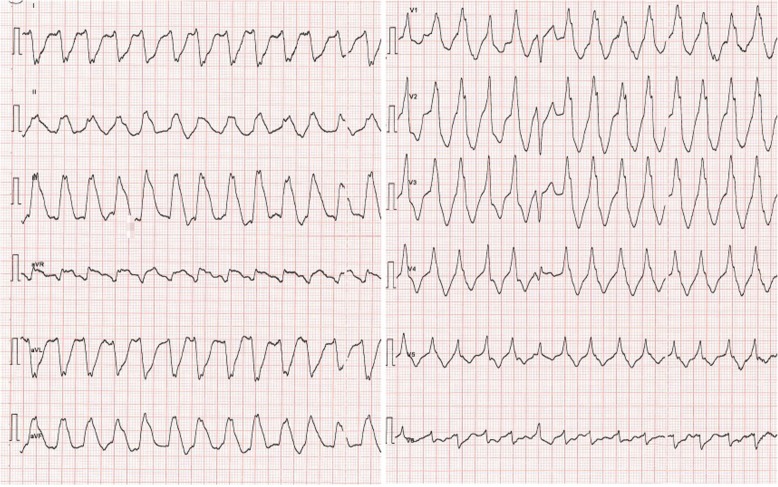
ECG: Ventricular tachycardia

**Fig. 4:**
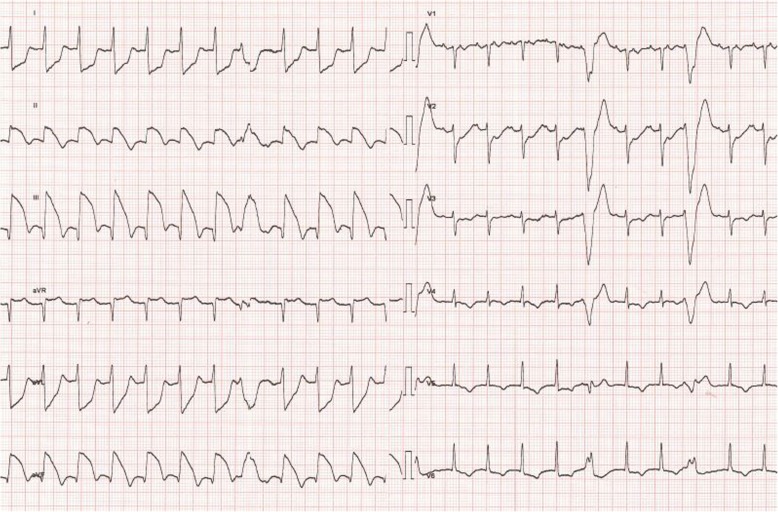
ECG: ST segment elevation in the inferior leads after cardioversion

**Fig. 5:**
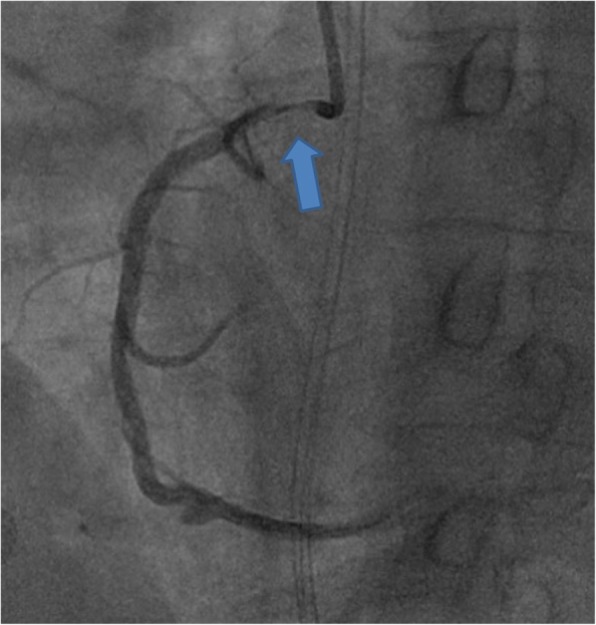
Angiography during right coronary artery vasospasm. Arrow indicates RCA vasospasm

In stable phase, during his fourth post-transplant week at the intermediate care unit, the patient developed sustained ventricular tachycardia at rest, with a heart rate of 150 bpm and no associated hemodynamic instability or even symptoms. Rapid cardioversion was easily achieved with a single administration of 2 mg magnesium sulfate IV. Nevertheless, the immediate post-cardioversion 12-lead ECG and echocardiography respectively showed severe ST-segment elevation in the inferior leads (Fig. 3 and 4) with inferior and inferolateral hypokinesia (Additional files 1, 2 and 3). Urgent cardiac catheterization showed a severe localized coronary vasospasm in the proximal RCA (Fig. 5), which was rapidly reversed after intracoronary injection of 1 mg isosorbide dinitrate. The ECG and echocardiography quickly normalized after the acute event, without consecutive elevation of troponins. An endomyocardial biopsy excluded an acute cellular or humoral rejection. The patient was treated with diltiazem without further episodes of arrhythmias or ST-segment changes during his hospital stay.

Interestingly, a few days before this coronary vasospasm, the patient complained from recurrent abdominal pain followed by melena. A colonoscopy showed ulcerations in the distal ileum, correlated with reversible signs of bowel ischemia in two consecutive CT scans, without evidence of occlusion or embolization in the mesenteric vessels. We could speculate that systemic factors in the recipient might favor vasospastic events. Because of a previously reported case of segmental mesenteric ischemia related to mycophenolate mofetil [17], this drug was replaced by azathioprine without any further abdominal pain or bleeding episodes.

## Discussion and conclusions

Three lessons can be learned from this case. First, vasospasm should never be forgotten as a possible cause of angiographic coronary stenosis, even in the setting of pre-transplant assessment: it may typically develop on the site of an atherosclerotic plaque, with the risk to overestimate the severity of a stenosis. In our case, this led to perform an unnecessary CABG in addition to transplant, which prolonged the ischemic time of these 54 years-old hearts over 3 h, and participated in the genesis of primary graft failure. The execution of provocation tests during the pre-transplant coronary angiogram could be considered for the early identification of this phenomenon in the donor heart: these tests have been proven to be not only safe but also useful to enhance the diagnostic value of a diagnostic angiography in the population suffering from vasospastic angina [18].

Secondly, it shows that coronary vasospasm may manifest very early in the recipient despite of complete autonomic denervation of the heart, and out of any context of graft rejection or cardiac graft vasculopathy. Although this was already reported [8], the particularity of our case is that a first coronary vasospasm could be documented in the donor during the pre-transplant coronary angiogram, which point out that this heart was sensitized to vasospasm in a context of preexistent atherosclerosis and probable endothelial dysfunction. Extensive ischemia-reperfusion injuries induced by the prolonged and severe primary graft failure and high doses of post-operative catecholamines may have exacerbated this dysfunction and favored vasospastic response.

Thirdly, our case shows that full heart recovery may still be possible after 9 days on ECMO support following primary graft failure with severe biventricular failure, which is longer compared to previously reported delays, usually ranging from 72 h to 7 days [19]. This could only be achieved through high-quality multidisciplinary care, which was life-saving for the patient. Whether CAS was also involved in primary graft failure in the immediate post-operative phase will never be known, though it has been reported elsewhere [14].

In conclusion, coronary vasospasm in heart-transplant patients is a complex and multi-faceted problem that is not limited to the context of graft rejection or cardiac graft vasculopathy. Independently of cardiac autonomic innervation, endothelial dysfunction - possibly induced by immunological, neurohormonal and metabolic factors – is now seen as the main trigger for this phenomenon, and may be exacerbated by ischemia-reperfusion injury in the near post-operative course. Our case identifies pre-existent atherosclerosis and vasospasm in the donor heart as a clear risk factor for early post-transplant vasospasm.

## Supplementary information

**Additional file 1.** Apical 4-chamber TTE view.

**Additional file 2.** Apical 2-chamber TTE view showing inferior hypokinesia.

**Additional file 3.** Apical 3-chamber TTE view showing infero-lateral hypokinesia.

## Data Availability

Not applicable.

## Abbreviations

Ach: Acethylcoline
CABG: Coronary artery bypass graft
CAS: Coronary artery spasm
CAV: Cardiac allograft vasculopathy
ECMO: Extra-corporeal membrane oxygenation
EM: Ergonovine maleate
IABP: Intra-aortic balloon pump
LVEF: Left ventricle ejection fraction
RCA: Right coronary artery
SPT: Spasm Provocating tests
